# Somatization symptoms and burnout: a correlational study among emergency nurses

**DOI:** 10.3389/fpubh.2025.1647123

**Published:** 2025-08-19

**Authors:** Ningxiang Li, Nan Xie, Xiaoli Chen, Hao Zhang, Luying Zhong, Dongmei Diao, Ling Zhu, Yue Zhou

**Affiliations:** ^1^Department of Emergency Medicine and West China School of Nursing, West China Hospital, Sichuan University, Chengdu, China; ^2^Disaster Medical Center, Sichuan University, Chengdu, China; ^3^Nursing Key Laboratory of Sichuan Province, Chengdu, China

**Keywords:** somatization symptoms, burnout, emergency nurse, mental health, sleep disturbances

## Abstract

**Background:**

Somatic symptom disorder is influenced by various factors, with increasing evidence highlighting its close association with burnout. This study aimed to investigate the correlation between somatization symptoms and burnout levels among emergency nurses, focusing on the impact of burnout on somatization.

**Methods:**

A cross-sectional study was conducted involving 1,540 emergency nurses working in tertiary hospitals in China. Data collection occurred between December 26, 2023, and January 18, 2024, using the Chinese versions of the Maslach burnout inventory–general survey (MBI-GS) and the somatic symptom disorder self-rating scale (SSD-CN). Statistical analyses were performed using SPSS version 25.0.

**Results:**

The mean SSD score was 39.58 ± 13.61, with 53.4% of participants exhibiting moderate to severe somatization symptoms. The mean burnout score was 4.77 ± 6.16, with a burnout incidence of 57.3%. A positive correlation was identified between the composite burnout score and the total SSD score (Pearson correlation coefficient = 0.534, *p* < 0.01). Emotional exhaustion and depersonalization scores were also positively correlated with individual SSD domain scores and the total SSD score (*p* < 0.01), Multivariate linear regression analysis revealed significant factors influencing somatization symptoms, including 6–10 and 11–15 years of work experience, weekly working hours of 41–48 or 49–58, 5–8 or >9 night shifts per week, monthly incomes ≥10,000 RMB and the composite burnout score (*p* < 0.05).

**Conclusion:**

Emergency nurses in this study exhibited severe somatization symptoms, with burnout identified as a significant contributing factor. To address this issue, healthcare management should consider implementing alternative shift patterns, optimizing workforce allocation, and revising compensation systems to ensure equitable labor distribution. These measures would support the healthy development of emergency nurses. Furthermore, nurses should prioritize self-care by engaging in activities such as psychological interventions, positive thinking training, and yoga to reduce burnout and enhance overall health.

## 1 Introduction

The occupational health of emergency nurses is significantly impacted by the urgency of their tasks, high workload, operational demands, and complex work environments. Somatic symptom disorder (SSD) is characterized by the presence of one or more somatic symptoms, often accompanied by mood disturbances, leading to severe anxiety, depression, and functional impairment in affected individuals (1). Among healthcare workers, the prevalence of SSD is reported to be 34.8% (2). A study conducted in the United States revealed that anxiety and depression are highly prevalent among healthcare workers, affecting over 80% of this population-substantially higher than in the general U.S. population (3). Nurses, in particular, experience elevated rates of anxiety and depression compared with other professions (4). The cooccurrence of increased somatic symptoms with heightened anxiety and depression intensifies the burden of illness among emergency nurses (5), underscoring the need for additional healthcare resources to support this workforce (6). As the largest professional group within the healthcare sector, nurses experiencing severe SSD are at risk of diminished care quality and heightened susceptibility to suicidal ideation (7). Despite the evident challenges, global studies on the health of emergency nurses remain limited. Therefore, comprehensive health surveys targeting emergency nurses are essential to establish a reliable foundation for improving clinical care management and promoting their health and wellbeing.

SSD is influenced by various factors, with increasing evidence highlighting its close association with burnout (8–11). Burnout is a syndrome characterized by emotional exhaustion, depersonalization, and a diminished sense of personal accomplishment, typically resulting from prolonged work-related stress (12). This negative emotional state adversely affects healthcare workers, leading to reduced work quality and efficiency, as well as increased conflicts with patients (13, 14). In 2019, the World Health Organization officially recognized burnout as an occupational health syndrome for the first time (15). Studies from multiple countries report burnout prevalence rates among healthcare workers reaching as high as 75% (16–18), with emergency nurses being disproportionately affected (19, 20), particularly in terms of emotional exhaustion (21). The nature of emergency nursing marked by high-risk, heavy workloads, and time sensitive tasks places these professionals at an elevated risk of burnout (22, 23). Research has demonstrated that burnout significantly impacts the mental health of healthcare workers, contributing to conditions such as anxiety, depression, cardiovascular disease and chronic pain (9, 10, 24–26). However, global studies in emergency medicine have provided limited evidence on the relationship between burnout and the health of emergency nurses. Therefore, exploring burnout levels and their health impact on this population warrants attention.

To address this gap, this study conducted a multicenter, large-sample, cross-sectional survey of emergency nurses across various regions of China. The study aimed to examine the correlation between somatization symptoms and burnout and to explore the impact of burnout on emergency nurses' health. The findings are intended to inform clinical interventions targeting burnout and promote health management strategies for emergency nurses, providing meaningful insights into mitigating this critical issue.

## 2 Methods

### 2.1 Research design

This study employed a cross-sectional survey design.

### 2.2 Survey respondents and sampling method

A stratified cluster sampling method was used to select participants. China was divided into seven geographic regions (Southwest, Northwest, South China, North China, East China, Central China, and Northeast China). From each region, two to three provincial capitals were chosen, one to two tertiary hospitals in each selected city were included, and a total of 30 hospitals were surveyed. Emergency nurses meeting the inclusion and exclusion criteria were enrolled in the study from December 26, 2023, to January 18, 2024. Inclusion criteria include at least 1 year of experience working in emergency medicine, licensed nurses employed in the emergency department, and aged 18 years or older. Exclusion criteria include nurses undergoing further training, specialist nurses, or those in standardized training programs, nurses with a previous medical diagnosis of a related psychiatric disorder, and nurses on sick leave, maternity leave, or lactation leave for 1 month or longer.

### 2.3 Research instruments

This survey collected data on general sociodemographic characteristics, somatization symptoms and burnout.

### 2.4 General sociodemographic information

Data were collected on the following variables: sex, education level, age, weekly working hours, night shifts, years of service, job title, parity and income.

### 2.5 Self-rating scale for somatic symptom disorder, Chinese (SSD-CN)

The Chinese version of the self-rating scale for somatic symptom disorder (SSD-CN) (27) was used. This scale consists of 20 items categorized into four domains: somatization symptoms, anxiety, depression, and combined anxiety-depression. The scale comprises 10 items assessing physical discomfort, four items evaluating anxiety, four items measuring depression, and two items addressing combined anxiety and depression. The scoring methodology assessed the occurrence and severity of each symptom on a scale from 1 to 4. A score of 1 indicated that the symptom was not present, 2 signified that it was present for a few days or tolerable, three indicated that it occurred for approximately half the days or was intermittently relieved, and four reflected symptoms that were present almost daily or difficult to endure. The total score ranged from 20 to 80. The severity of SSD was classified as follows: 20–29 was considered normal, 30–39 indicated mild severity, 40–59 represented moderate severity, and scores of 60 or higher were categorized as severe. Correlation coefficients for the SSD scale ranged from 0.76 to 0.88 between the domains and the total score and from 0.56 to 0.70 within each domain, demonstrating strong internal consistency. The test–retest reliability of the scale was 0.9. A cutoff score of 36 was established for diagnostic purposes, with a sensitivity of 0.97 and a specificity of 0.96.

### 2.6 Burnout survey

Burnout was evaluated using the Chinese version of the Maslach burnout inventory-general survey, adapted by Wei et al. (28). It is used to assess burnout levels among emergency nurses. The scale, validated by Chinese scholars Yueping et al. (29) demonstrated good reliability and validity. The MBI-GS comprises 16 items scored on a seven-point Likert scale ranging from 0 to 6. These items are divided into three domains: emotional exhaustion, depersonalization, and work achievement. Emotional exhaustion and depersonalization scores were interpreted positively, with higher scores indicating greater burnout. Conversely, the work achievement domain was scored negatively, with lower scores signifying greater burnout. A burnout score of <1.5 indicated no burnout, a score between 1.5 and 3.5 suggested suspected burnout, and a score of 3.5 or higher confirmed burnout. Cronbach's α coefficients for the overall scale and its three domains were 0.860, 0.914, 0.814, and 0.899, respectively, demonstrating good internal consistency.

### 2.7 Data collection

Data were collected using an electronic version of the questionnaire. To ensure completeness and prevent duplication, all questions were set as mandatory and could only be answered once by each participant. One investigator at each hospital provided training on completing the questionnaire before the survey. The survey link was distributed via the WeChat platform, and the study's purpose was explained to participants. Participation was anonymous, and respondents were required to read and provide consent via an informed consent form before accessing the questionnaire. Once completed, respondents submitted their responses.

### 2.8 Methods of statistical analysis

Data were imported into SPSS 25.0 for statistical analysis frequency and percentage were used to describe categorical data, while the mean and standard deviation were used for continuous data. Analysis of variance was employed to compare SSD scores and overall burnout scores across demographic characteristics. Pearson's was used to analyse studies on the correlational relationship between somatisation symptoms and burnout, and multivariate linear regression was applied to assess the effects of somatization symptoms and burnout on each other.

## 3 Results

### 3.1 Basic information

A total of 1,550 questionnaires were collected, of which 1,540 were valid after excluding 10 with identical responses. Among the participants, 1,211 (78.6%) were female, and 329 (21.4%) were male. The majority of participants (734, or 47.7%) were aged 30–39 years. Regarding educational background, 1,354 participants (87.9%) held a bachelor's degree or higher. Half of the respondents (780, or 50.6%) worked 41–48 h per week, and 1,359 participants (88.2%) reported working night shifts, as shown in Table 1.

**Table 1:** General demographic information.

**Variables**	**Otcategory**	**Frequency (*n*)**	**Percent (%)**
Age	20–29 years	582	37.8
	30–39 years	734	47.7
	40–49 years	183	11.9
	≥50 years	41	2.7
Gender	Female	1,211	78.6
	Male	329	21.4
Marital status	Unmarried	560	36.4
	Married	980	63.6
Fertility status	Yes	710	46.1
	No	830	53.9
Education	Occupation school	186	12.1
	Bachelor's or higher degree	1,354	87.9
Job title	Junior	907	58.9
	Intermediate	574	37.3
	Senior	59	3.8
Work experience	≤ 5 years	491	31.9
	6–10 years	493	32.0
	11–15 years	300	19.5
	16–20 years	121	7.8
	>20 years	135	8.8
Working hours per week	<40 h	577	37.5
	41–48 h	780	50.6
	49–58 h	123	8.0
	≥59 h	60	3.9
Night shift frequency	0/month	181	11.8
	1–4/month	239	15.5
	5–8/month	659	42.8
	≥9/month	461	29.9
Salary (CNY)	<4,000 yuan	57	3.7
	4,000–5,999 yuan	149	9.7
	6,000–7,999 yuan	250	16.2
	8,000–9,999 yuan	368	23.9
	≥10,000 yuan	716	46.5

### 3.2 SSD score and overall burnout score analysis

The total SSD score was 39.58 ± 13.61, with 53.4% of participants experiencing moderate to severe somatic symptoms. Only 25.8% reported no somatic symptoms, as shown in Table 2. The overall burnout score was 4.77 ± 6.16, with burnout affecting 57.3% of participants and suspected burnout observed in 21.2%, as shown in Table 3.

**Table 2:** Descriptive statistics of somatization symptom severity.

**Item**	**Frequency**	**Percent (%)**
Considered normal (20–29)	397	25.8
Indicated mild severity (30–39)	320	20.8
Represented moderate severity (40–59)	664	43.1
Higher were categorized as severe (≥60)	159	10.3

**Table 3:** Descriptive statistics of burnout severity.

**Item**	**Frequency**	**Percent (%)**
Indicated no burnout <1.5	331	21.5
Suspected burnout 1.5–3.5	327	21.2
Higher confirmed burnout ≥3.5	882	57.3

### 3.3 Two-way correlation analysis of burnout and SSD scores by domain

The overall burnout score showed a significant positive correlation with the total SSD score (Pearson's correlation coefficient: 0.534, *p* < 0.01). The emotional exhaustion scores were both significantly positively correlated with each SSD domain and the total SSD score (Pearson's correlation coefficient:0.575, 0.591, 0.642, 0.569, 0.623, *p* < 0.01), depersonalization domain scores were both significantly positively correlated with each SSD domain and the total SSD score (Pearson's correlation coefficient: 0.584, 0.603, 0.624, 0.550, 0.624, *p* < 0.01). However, the work achievement domain was not correlated with any SSD domain, as shown in Table 4.

**Table 4:** Pairwise correlation analysis of burnout and SSD scores by domain.

**Item**	**Burnout**				**SSD-CN**				
	**Emotional exhaustion**	**Depersonalization**	**Work achievement**	**Score**	**Somatization symptoms**	**Anxiety**	**Depression**	**Combined anxiety-depression**	**Score**
1	1								
2	0.878^**^	1							
3	−0.096^**^	0.013	1						
4	0.876^**^	0.806^**^	−0.533^**^	1					
5	0.575^**^	0.584^**^	0.014	0.498^**^	1				
6	0.591^**^	0.603^**^	0.035	0.504^**^	0.901^**^	1			
7	0.642^**^	0.624^**^	0.037	0.536^**^	0.848^**^	0.867^**^	1		
8	0.569^**^	0.550^**^	−0.014	0.497^**^	0.794^**^	0.788^**^	0.868^**^	1	
9	0.623^**^	0.624^**^	0.021	0.534^**^	0.975^**^	0.950^**^	0.933^**^	0.875^**^	1

^**^p < 0.01, the correlation was significant.

### 3.4 Factors influencing somatization symptoms univariate analysis

The univariate analysis revealed that gender, age, marital status, reproductive status, weekly working hours, frequency of night shifts, job title, monthly income, years of service, and education were all statistically significant factors influencing somatization symptoms (*p* < 0.05), as shown in Table 5.

**Table 5:** Univariate analysis of factors affecting somatization symptoms.

**Variables**	**Category**	**Score $\bar{x}\pm s$**	***t*/*F***	***p*-Value**
Age	20–29 years	36.71 ± 13.59	15.489	0.000
	30–39 years	41.31 ± 13.45		
	40–49 years	42.19 ± 13.01		
	≥50 years	37.88 ± 12.33		
Gender	Female	39.96 ± 13.45	2.082	0.038
	Male	38.20 ± 14.15		
Marital status	Unmarried	37.95 ± 14.11	−3.521	0.000
	Married	40.52 ± 13.23		
Fertility status	Yes	38.03 ± 13.75	−4.156	0.000
	No	40.91 ± 13.36		
Education	Occupation school	36.34 ± 12.91	−3.475	0.001
	Bachelor's or higher degree	40.03 ± 13.65		
Job title	Junior	37.82 ± 13.56	19.012	0.000
	Intermediate	42.11 ± 13.16		
	Senior	42.12 ± 14.63		
Work experience	≤ 5 years	36.22 ± 13.42	11.803	0.000
	6–10 years	40.69 ± 13.90		
	11–15 years	41.65 ± 13.33		
	16–20 years	42.22 ± 12.09		
	>20 years	40.86 ± 13.03		
Working hours per week	<40 h	37.24 ± 12.95	13.411	0.000
	41–48 h	40.29 ± 12.90		
	49–58 h	43.24 ± 16.12		
	≥59 h	45.47 ± 18.39		
Night shift frequency	0/month	38.66 ± 11.91	8.919	0.000
	1–4/month	36.02 ± 12.09		
	5–8/month	39.80 ± 13.52		
	≥9/month	41.48 ± 14.72		
Salary (CNY)	<4,000 yuan	43.09 ± 17.69	2.720	0.028
	4,000–5,999 yuan	41.13 ± 16.29		
	6,000–7,999 yuan	40.27 ± 14.91		
	8,000–9,999 yuan	39.98 ± 12.85		
	≥10,000 yuan	38.54 ± 12.42		

### 3.5 Multivariate analysis

Multivariate linear regression analysis identified several factors influencing somatization symptoms. These included 6–10 years of service (*B* = 2.914; 95% CI, 0.542–5.286) and 11–15 years of service (*B* = 3.374; 95% CI, 0.413–6.335), weekly working hours of 41–48 h (*B* = 1.443; 95% CI, 0.217–2.669) and 49–58 h (*B* = 2.524; 95% CI, 0.291–4.756), 5–8 night shifts per week (*B* = 2.294; 95% CI, 0.200–4.389) and ≥9 night shifts per week (*B* = 2.575; 95% CI, 0.394–4.755), a monthly income ≥10,000 RMB (*B* = −5.598; 95% CI, −8.787 to −2.408), and burnout (*B* = 1.092; 95% CI, 0.997–1.187), all of which were statistically significant (*p* < 0.05), as shown in Table 6.

**Table 6:** Multivariate linear regression analysis of factors affecting somatization symptoms.

**Variables**	**Category**	**SE**	**β**	***t***	***p*-Value**	**95% CI**	
						**LLCI**	**ULCI**
Age	20–29 years						
	30–39 years	1.231	0.047	1.047	0.295	−1.126	3.703
	40–49 years	2.082	0.036	0.728	0.467	−2.568	5.601
	≥50 years	2.971	−0.009	−0.255	0.799	−6.585	5.070
Gender	Female						
	Male	0.727	−0.017	−0.794	0.427	−2.004	0.849
Marital status	Unmarried						
	Married	1.038	−0.060	−1.637	0.102	−3.736	0.337
Fertility status	Yes						
	No	1.036	0.035	0.931	0.352	−1.068	2.997
Education	Occupation school						
	Bachelor's or higher degree	0.927	0.015	0.660	0.509	−1.206	2.430
Job title	Junior						
	Intermediate	0.775	0.045	1.637	0.102	−0.251	2.789
	Senior	1.790	0.001	0.033	0.974	−3.452	3.569
Work experience	≤ 5 years						
	6–10 years	1.209	0.100	2.410	0.016^*^	0.542	5.286
	11–15 years	1.510	0.098	2.235	0.026^*^	0.413	6.335
	16–20 years	1.988	0.072	1.834	0.067	−0.254	7.546
	>20 years	2.491	0.076	1.468	0.142	−1.229	8.542
Working hours per week	<40 h						
	41–48 h	0.625	0.053	2.309	0.021^*^	0.217	2.669
	49–58 h	1.138	0.050	2.218	0.027^*^	0.291	4.756
	≥59 h	1.575	0.031	1.390	0.165	−0.900	5.280
Night shift frequency	0/month						
	1–4/month	1.198	−0.010	−0.322	0.748	−2.735	1.964
	5–8/month	1.068	0.083	2.149	0.032^*^	0.200	4.389
	≥9/month	1.111	0.087	2.316	0.021^*^	0.394	4.755
Salary (CNY)	<4,000 yuan						
	4,000–5,999 yuan	1.777	−0.022	−0.558	0.577	−4.477	2.493
	6,000–7,999 yuan	1.694	−0.072	−1.559	0.119	−5.964	0.682
	8,000–9,999 yuan	1.652	−0.091	−1.766	0.078	−6.158	0.323
	≥10,000 yuan	1.626	−0.205	−3.443	0.001^*^	−8.787	−2.408
Burnout	Score	0.048	0.494	22.550	0.000^*^	0.997	1.187

SE, standard error; C, confidence internal; LLCI, lower limit confidence interval; ULCI, upper limit confidence interval.. ^**^P < 0.01, ^*^P < 0.05.

## 4 Discussion

The study found that severe somatization symptoms affected 53.4% of emergency nurses, underscoring the urgent need to improve their health. These findings align with other studies indicating high levels of anxiety, depression, and severe somatization symptoms among healthcare workers (2, 30, 31). The continuous development of the medical industry has resulted in an increasingly heavy workload for emergency nurses, who are expected to possess advanced professional skills and scientific research capabilities. Emergency nurses are frequently exposed to a range of traumatic situations and accidents, often confronting death, which can lead to posttraumatic stress disorder and increase the risk of mental health issues and somatization symptoms. To address these challenges, management can introduce online information management methods aimed at reducing depression and anxiety (4), such as text message reminders, mobile mental health assessments, and online continuity of care programs. Furthermore, emotional awareness and expressive arts therapy have been shown to significantly reduce somatic symptoms (32). Our survey indicates a high prevalence of burnout among emergency nurses in China, with an incidence rate of 57.3%. This finding aligns with previous studies (33–36), which also highlighted a high level of burnout among emergency nurses. However, this result contrasts with a study by Adriaenssens et al. It could be that the career is too stressful and while excessive effort increased the risk of burnout (37, 38) which reported a burnout prevalence of 26% among emergency nurses in Western countries. This discrepancy may stem from differences in healthcare systems and medical environments. Western countries generally have more robust healthcare resources and more equitable access to care, while Chinese tertiary hospitals face challenges such as uneven medical resources, overcrowded facilities, high patient expectations, which contribute to significant pressure and burnout among emergency nurses (36). Improve working conditions, such as developing flexible workload management, increasing salaries, adjusting promotion mechanisms, and supporting policies to alleviate burnout (37). Personalized music interventions have been recommended as an adjunctive method to alleviate emotional exhaustion among emergency nurses, thus enhancing their overall health (33).

The correlation analysis in this study demonstrated that the overall burnout score was positively correlated with the total SSD score, meaning that higher burnout scores were associated with more severe somatization symptoms. This finding is consistent with other studies, which have shown that burnout syndrome is significantly linked to higher rates of depression and somatic disorders (39, 40). The results of the multivariate hierarchical regression analysis indicated that burnout was a significant factor influencing somatization symptoms. This finding is consistent with several studies that have concluded burnout negatively impacts health (14, 26), serving as a risk factor for musculoskeletal pain, cardiovascular disease, and depression (9, 11). Gulen et al. (42) suggested that both depression and burnout syndromes are influenced by S100B protein levels. Similarly, Pilger et al. (43) found that salivary cortisol levels influenced anxiety and depressive symptoms, which could be treated to alleviate the health status of nurses. Long-term exposure to noisy environments in Chinese emergency departments activates the sympathetic nervous system, inducing a stress response (34). Emergency nurses face common occupational health problems, and experiences of workplace violence can have a serious impact on their physical and mental health (44). In addition to their ability to manage emergencies and apply their nursing skills, emergency nurses must also cope with pressure from patients and their families. Working in such a complex environment can lead to burnout, anxiety, depression, and somatic discomfort. Flexible work schedules, telecommuting options, and family support policies should be introduced that may help increase job satisfaction and reduce burnout (45, 46). Haghighinejad et al. (47) concluded that positive thinking training could be an effective strategy to reduce burnout and improve health. The researchers (41, 48) found that yoga training led to significant improvements in both burnout and overall health, recommending that nurses engage in such practices during their spare time. Medisauskaite et al. (49) highlighted that education in psychological knowledge significantly reduced anxiety. The harm of workplace violence to the health of emergency nurses can be mitigated by improving sleep quality (44). Effective nurse management can further alleviate burnout by rationally allocating human resources, providing diverse promotional opportunities, and optimizing the work environment to enhance motivation. This would not only improve the overall health of emergency nurses but also reduce burnout levels.

The results of the multivariate hierarchical regression analysis revealed that several factors such as having 6–15 years of work experience, working 41–58 h per week, working more than five night shifts per week, and earning a monthly income of ≥10,000 RMB significantly influenced somatization symptoms. Emergency nurses with 6–15 years of experience make up 51.5% of the workforce, serving as the backbone of emergency clinical care. However, they are often burdened with family caregiving responsibilities, which, when combined with the demands of their profession, can lead to work-family role behavioral conflicts, negatively impacting both their physical and mental health (50, 51). Therefore, it is crucial for mid-career and senior emergency nurses to engage in research and innovation, contribute new knowledge and technologies to clinical practice, and find ways to balance work with family obligations, thereby enhancing their professional value and sense of accomplishment. Extended working hours and frequent night shifts can cause fatigue, disrupt circadian rhythms, and impair sleep. making it difficult for their bodily functions to adapt to the shift system, leading to sleep deprivation, which in turn affects the clearance of β-amyloid from the brain, and then causes sleep disorders. Sleep disturbances can have a serious negative impact on nurse health, patient safety, and social productivity (52, 53). Improved staffing levels and the ability to take uninterrupted breaks have been shown to positively affect the health status of emergency nurses (54). Management should explore alternative shift patterns (44), reduce the frequency of night shifts, and ensure sufficient rest periods to mitigate burnout and improve the nurses' occupational health. Finally, monthly income ≥10,000 RMB was found to be a protective factor against somatization symptoms. Management should consider ensuring that labor compensation is distributed fairly and reasonably, as this can play a key role in promoting the health and wellbeing of emergency nurses (37).

## 5 Limitations

This study only examined the correlation between burnout and somatization symptoms, without establishing a causal relationship. Future longitudinal studies may explore this relationship in greater depth. Additionally, the study population, environment, and job characteristics are specific to emergency nurses and may not be representative of other occupational groups. Further research is needed to determine the generalizability of these findings to other fields. Finally, the use of self-reported measures, which may introduce response or recall bias.

## 6 Conclusions

This study found that emergency nurses experience significant somatization symptoms, with their overall burnout score being positively correlated with the total SSD score. Somatization symptoms were influenced by multiple factors, with burnout identified as a key determinant. This was determined through a multicenter, large-sample survey. Management should prioritize exploring alternative shift patterns, optimizing human resource allocation, and refining the compensation system. At the individual level, strategies such as positive thinking training, yoga, and personalized music interventions can help reduce burnout and improve the health of emergency nurses.

## Data Availability

The original contributions presented in the study are included in the article/supplementary material, further inquiries can be directed to the corresponding author.
